# Relevant parameters for recommendations of physical activity in patients suffering from multiple myeloma: A pilot study

**DOI:** 10.1007/s00508-019-01582-z

**Published:** 2019-11-29

**Authors:** Fadime Cenik, Mohammad Keilani, Timothy Hasenöhrl, Dominikus Huber, Bianca Stuhlpfarrer, Anna Pataraia, Richard Crevenna

**Affiliations:** Department of Physical Medicine, Rehabilitation and Occupational Medicine, Medical University of Vienna, Waehringer Gürtel 18–20, 1090 Vienna, Austria

**Keywords:** Plasmacytoma, Exercise, Self-help groups, Bone lesion

## Abstract

**Purpose:**

This pilot study aimed to describe physical performance, self-reported physical activity, health-related quality of life, anxiety and depression in patients who were assigned from Austrian self-help groups for multiple myeloma patients. These parameters were then discussed in the context of clinical decision-making concerning the recommended type of regular physical activity and exercise.

**Methods:**

Members of the self-help groups were invited to participate. Physical performance and physical activity were assessed with the 6 min walk test (6MWT), handgrip strength test, timed up and go test (TUG), Tinetti performance oriented mobility assessment (POMA), falls efficacy scale (FES), international physical activity questionnaire (IPAQ), health-related quality of life (EORTC QLQ-C30) and the hospital anxiety and depression scale (HADS).

**Results:**

A total of 40 patients (female:male = 15:25, mean age: 63.8 ± 9.0 years, range 41–80 years) were identified. In total 20 (50%) reached the performance of healthy peers in the tests 6MWT, handgrip strength, TUG and POMA, while 50% showed at least 1 result below the reference value or cut-off-point for each test. Self-reported activity levels were high. Patients showed a tendency to overestimate the risk of falling but a case by case analysis revealed a tendency for underestimating the actual performance in the respective tests (TUG, POMA).

**Conclusion:**

The performance of healthy peers was reached by a substantial number of the participants in tests of physical performance and they reported high levels of physical activity. Nevertheless, they tended to overestimate the specific risk of falling. Patients with notably impaired physical performance might be suitable to perform regular physical activity and exercise in an individual therapy, whereas those with good physical performance are suited for training in exercise groups; however, individual contraindications and clinical considerations should be noted in a multiprofessional and interdisciplinary setting.

## Introduction

Multiple myeloma is the second most common hematologic malignancy [1]. Patients suffering from multiple myeloma show an increase in cancer-specific survival rate due to modern cancer treatment [2, 3]. Regular physical activity and exercise are important measures to improve health-related quality of life and physical performance in cancer patients [4–7]. Recommendations for regular physical activity and exercise consist of 150 min/week of moderately intense or 75 min/week of vigorously intense activity or an equivalent combination, muscle strengthening activities of at least moderate intensity at least 2 days/week for each major muscle group and stretching of major muscle groups and tendons [2, 4–12].

In Austria approximately 2200 patients suffer from multiple myeloma [13]. There are two main self-help groups in Austria, the so-called *Multiples Myelom* and *Myelom- und Lymphomhilfe* offering regular meetings and conferences for patients [14, 15]. Nevertheless, the opportunity to be involved in myeloma exercise groups does not yet exist in Austria. Due to bone manifestation of multiple myeloma and special clinical features (especially hypercalcemia and monoclonal gammopathy), a multiprofessional and interdisciplinary approach is required for planning and prescribing regular physical activity and exercise [2, 8–12]. Hereby, physical performance seems to be an important factor to identify which patients should receive an individual therapy or a therapy in an exercise group [11, 16–19]. Physical performance depends on endurance capacity, muscle strength and sensorimotor function.

This pilot study aimed to describe physical performance, self-reported physical activity, health-related quality of life, anxiety and depression in patients who were assigned from two Austrian self-help groups. These parameters were then discussed in the context of clinical decision-making, concerning the recommended type of regular physical activity and exercise.

## Patients, materials and methods

The two main self-help groups (*Myelom- und Lymphomhilfe* and *Multiples Myelom*) invited their members with multiple myeloma (aged between 18 and 99 years) to participate in this cross-sectional study. The patients were invited via conferences, emails and the webpage and also via Facebook. After agreeing to participate and checking out the inclusion criteria via telephone, a medical examination was carried out to survey status and demographic data before assessing the parameters presented below. Patients with preliminary stages, such as monoclonal gammopathy of undetermined significance (MGUS) or asymptomatic (smoldering/indolent) myeloma without targeted cancer treatment were excluded from this study. Furthermore, the patients who did not fully understand the study measures or could not perform them, did not speak German sufficiently well or were unable to cognitively or physically follow the course of study were excluded. Illiteracy and refusal to participate were other exclusion criteria. To give the patients from all regions of Austria the best opportunity to participate in this study travel expenses were remunerated.

This pilot study took place at the Department of Physical Medicine, Rehabilitation and Occupational Medicine of the Medical University of Vienna, Austria. The ethics committee of the Medical University of Vienna, Austria approved the present study (EK.-Nr.: 1725/2018). All patients signed a written informed consent before enrolment. The participants were assessed from February 2019 to July 2019.

### Assessment

To assess physical performance, the 6‑min walk test (6MWT), the handgrip strength test (HGST by JAMAR® dynamometry [Patterson Medical, Warrenville, IL, USA]), the timed up and go test (TUG) and the Tinetti performance oriented mobility assessment (POMA) were performed. Additionally, the patients were asked to fill in a series of standardized questionnaires and scales:

- Health-related quality of life of the participants (EORTC QLQ-C30)
- Clinically significant states of anxiety and depression (hospital anxiety and depression scale, HADS)
- Self-reported physical activity (international physical activity questionnaire, IPAQ)
- Fall-related self-efficacy (falls efficacy scale, FES)

The TUG is a commonly used screening tool to assist clinicians to evaluate gait and balance functions, and to identify patients at risk of falling [20]. There are different procedures for the TUG, namely with normal and fast walking speeds [20]. In the present study the normal walking speed and cut-off-point for impaired mobility were used (>10 s) based on the recommendations of the Austrian Society for Geriatrics and Gerontology [21] as well as Podsiadlo and Richardson [22]. Concerning the POMA, the assessment was based on Tinetti with the following classification: <19 points for high fall risk, 19–23 points for moderate fall risk and 24–28 points for minimal fall risk [23].

The IPAQ was used to indirectly evaluate an individual’s extent of sedentary behavior and moderate to vigorous physical activity throughout the last 7‑day week. The questionnaire was scored in terms of estimated metabolic equivalent task minutes per week (MET-min/week) [24]. For the FES the cut-off-points established by Delbaere et al. were used, who classified low concern about falling with 16–19 points, moderate concern with 20–27 points and high concern with 28–64 points [25]. The HADS evaluation was based on Zigmond and Snaith with the following classification and definition: 0–7 points as normal case, 8–10 as doubtful case, ≥11 as definitive case for each sub-scale [26]. The age-matched normative data published by Breeman et al. were used for analysis, which were available only for people younger than 65 years old [27]. Therefore, all older participants were classified as being 65 years old in this study. The 6MWT is widely used as a test of general physical performance, mobility and submaximal exercise capacity [28]. For the 6MWT, the following formula was used to calculate the predicated walking distance in meters for each participant: (7.57×height_cm_) − (5.02×age_year_) − (1.76×weight_kg_) − 309 for male and (2.11×height_cm_) − (2.29×age_year_) − (5.78×weight_kg_) + 667 for female participants [28]. For the HGST mean values of each side were used to compare with the JAMAR® age and sex-matched reference values [29–31]. Existing reference values with multiple myeloma patients from the EORTC group were taken from Scott et al. (2008) [32]. Continuous variables are presented as means and standard deviations, medians and ranges and nominal variables as percentages. The Kolmogorov-Smirnov test was used to test each variable for normal distribution. In case the null-hypothesis from this test was retained, non-parametric solutions like Wilcoxon-signed rank test and Spearman’s correlation were employed to test the related samples for difference in means and correlation, respectively. Due to the retrospective character of the study, no risk ratios could be calculated. Nonetheless, odds ratios (OR, corrected via Haldane-Anscombe for low sample size) were calculated for a pathological test result in one of the primary outcome parameters (columns) given a specific patient characteristic (rows).

## Results

In total, 55 patients agreed to participate of whom 9 were excluded due to exclusion criteria (suffering from preliminary stages; had not received any cancer treatment), 4 were not available after self-registration for this study, 1 patient did not want to travel to the study location and 1 patient was recommended not to participate by the family physician. Finally, 40 patients (female:male = 15:25) fulfilled the inclusion criteria. The demographic data of the participants are presented in Table 1.

**Table 1:** Demographic data

***n*** **=40**	**Mean**	**SD**	**Range**
*Age (years)*	63.8	9.0	41–80
Female	64.5	7.7	52–80
Male	63.3	9.8	41–80
*Family status*	*n*		
Married	23		
Cohabitation	6		
Divorced	4		
Single	3		
Widowed	3		
n. s.	1		
*Employment*			
Yes	7		
No (retired)	32		
No (housewife)	1		
*Highest level of education*			
University	13		
Business/high school	15		
Obligatory education/apprenticeship	12		
*Household income/year *(€)			
10,000–25,000	10		
25,000–50,000	17		
>50,000	3		
n. s.	10		
*Smoker*	3		
*Time since initial diagnosis*			
<1 year	5		
1–2 years	5		
3–5 years	10		
5–10 years	12		
>10 years	8		
*Stem cell transplantation*			
Yes	28		
No	12		
*Pathologic fracture/risk*			
Yes	21		
No	19		
*Radiation due to fracture/risk*			
No	21		
Yes	19		
*Operation due to fracture/risk*			
No	31		
Yes	9		
*Polyneuropathy*	22		
*Cancer rehabilitation performed*			
No	20		
Yes	19		
n. s.	1		

*SD* standard deviation, *n.* *s.* not specified

Each patient had received (*n* = 8) or was still receiving (*n* = 32) chemotherapy, new substances and/or immunotherapy (with or without stem cell transplantation). The results of the 6MWT, HGST, POMA, TUG, HADS, IPAQ and EORTC QLQ-C30 are presented in Table 2.

**Table 2:** Results of all parameters

**Parameter**	**Total (*n*** **=40)** **mean** **±SD**	**Median**	**Range**
*6‑MWT in m*	548 ± 128	573	225–835
*HGST in kg*	32.4 ± 9.6	32.5	17–51
*POMA (<19* *=high fall risk)* (points)	23.5 ± 4.8	26	12–28
Balance	13.4 ± 3.0	15	5–16
Gait	10.1 ± 2.1	11	6–12
*TUG (s)*	9.5 ± 3.1	9	5.5–21.0
*FES*	21.2 ± 6.0	20	16–39
*HADS* (points)	10.5 ± 5.0	10	1–23
Anxiety	5.7 ± 3.2	6	0–12
Depression	4.9 ± 2.7	4	1–11
*MET-min/week*	**Total (*n*** **=35)** **mean** **±SD**		
Vigorous	888.0 ± 1873.3	0	0–10,080
Moderate	830 ± 1275.3	240	0–5040
Walk	1110.7 ± 1127.8	792	0–2772
Total	2829.5 ± 3041.5	1584	0–16,506
*EORTC QLQ-C30* (points)	**Total (*n*** **=40)** **mean** **±SD**		**Reference**
Global health status/QOL	60.0 ± 21.5	62.5	55.7 ± 22.8
Physical functioning	75.3 ± 18.1	80.0	67.7 ± 23.4
Role functioning	65.0 ± 23.5	66.7	60.1 ± 33.4
Emotional functioning	67.3 ± 24.7	66.7	71.3 ± 22.7
Cognitive functioning	70.8 ± 24.7	66.7	78.1 ± 23.8
Social functioning	64.6 ± 29.0	66.7	63.2 ± 31.0
Fatigue	47.8 ± 22.3	44.4	48.7 ± 26.7
Nausea and vomiting	7.1 ± 13.5	0	10.5 ± 19.2
Pain	36.7 ± 30.5	33.3	47.1 ± 33.9
Dyspnea	30.0 ± 25.9	33.3	26.0 ± 27.3
Insomnia	42.5 ± 33.7	33.3	28.9 ± 30.6
Appetite loss	12.5 ± 24.7	0	23.2 ± 30.2
Constipation	13.3 ± 27.0	0	23.2 ± 29.9
Diarrhea	20.0 ± 30.0	0	9.6 ± 19.4
Financial difficulties	20.0 ± 29.0	0	16.1 ± 26.6

*SD* standard deviation, *QOL* quality of life, *6MWT* 6-min walk test, *HGST* handgrip strength test, *POMA* Tinetti performance oriented mobility assessment, *TUG* timed up and go test, *FES* falls efficacy scale, *HADS* hospital anxiety and depression scale, *MET* metabolic equivalent of task, *EORTC QLQ-C30* European Organization for Research and Treatment of Cancer Quality of Life Questionnaire-Core 30

On average the participants showed a moderate subjective risk of falling (moderate concern in the FES) and a low to moderate fall risk in the objective fall risk assessments (POMA and TUG test, Table 3). In more detail, 20 patients (50%) reported a moderate or high concern about falling (FES >10) while in fact only 10 patients (25%) showed an elevated risk in the TUG test, of whom 9 patients (22.5%) were deemed at high risk by the POMA. Among those patients who reported a moderate to high subjective risk of falling (in the FES), 70% performed well in the remaining objective risk assessment tools. A case by case analysis therefore revealed a tendency of patients to overestimate the fall risk and underestimate the actual performance in the respective tests.

**Table 3:** Patients underwent four different objective measures of physical performance (6MWT, POMA, TUG, HGST)

Sex	Age (years)	6MWT (m)	HGST (kg)	POMA (points)	TUG (s)
m	59	*600*	*38*	*28*	*8*
m	54	*643*	*44*	*28*	*7*
m	61	*640*	*41*	*27*	*7*
f	67	*530*	*26*	*25*	*9*
m	70	*550*	*38*	*27*	*8*
m	65	*750*	*42*	*28*	*6*
f	64	*475*	*26*	*27*	*9*
f	59	*560*	*26*	*27*	*9*
f	63	*580*	*34*	*28*	*9*
m	67	*602*	*51*	*28*	*8*
f	52	*625*	*30*	*27*	*8*
f	66	*605*	*22*	*26*	*8*
f	66	*517*	*32*	*26*	*7*
m	51	*720*	*45*	*27*	*9*
m	60	*605*	*43*	*26*	*9*
m	55	*835*	*40*	*26*	*8*
m	70	*620*	*30*	*26*	*7*
m	57	*660*	*50*	*28*	*9*
m	70	*680*	*34*	*27*	*6*
f	61	*575*	*26*	*28*	*7*
m	69	**470**	36	26	7
m	59	600	50	**20**	8
f	66	460	22	**22**	8
m	79	500	26	**16**	9
m	61	**500**	32	25	10
f	62	590	**15**	24	9
m	53	650	**40**	27	8
f	57	**570**	26	24	**13**
f	71	**478**	18	**21**	9
m	78	525	30	**18**	10
m	55	660	**20**	25	**13**
m	72	520	35	**18**	**11**
m	41	**650**	**36**	**21**	9
m	59	**358**	**30**	**22**	**12**
f	78	**360**	20	**16**	**14**
f	55	**305**	**17**	**15**	**12**
m	61	**425**	38	**13**	**11**
m	80	**427**	50	**18**	**11**
m	77	**225**	37	**12**	**21**
f	80	**270**	18	**15**	**19**

*N* = 20 patients showed a pathological result in at least one test (*bold*) while the other 20 patients reached the individual cut-off-points of the healthy peers (*italic*)

*m* male, *f* female, *6MWT* 6-min walk test in meters, *POMA* Tinetti performance oriented mobility assessment, *TUG* timed up and go test, *HGST* handgrip strength test of the dominant hand in kilograms

The third objective test for physical performance was the handgrip strength test. Overall, 6 (15%) participants showed a reduced handgrip strength in comparison with age and sex-matched reference values. The 6MWT was employed as fourth objective evaluation of physical performance and 12 patients showed results below the individual age, sex, height and weight-predicted values (see methods). Results from this test are the only data in this study that followed a normal distribution (Kolmogorov-Smirnov test retains the null hypothesis of normal distribution at *p* > 0.20). Patients walked on average 547.9 m in 6 min and the standard deviation was 127.8 m. Patients who did not reach the predicted value (*n* = 12) walked on average 419 m and those who did reach the predicted values (*n* = 28) 603 m. Despite the low sample size, both groups followed a normal distribution (Kolmogorov-Smirnov test *p* > 0.20) and the means in these two groups are highly significantly different (2-sided Wilcoxon-signed-rank test *p* < 0.01). On average, patients who reached the predicted values walked 43% further in 6 min than the peers who did not reach their predicted values. When combining the objective tests for hand grip strength, exercise capacity (6MWT) and risk of falling (TUG test and POMA), 20 (50%) patients in the sample of 40 multiple myeloma patients showed a pathologic result in at least 1 test, while the other 50% performed above the cut-off points in all tests (Table 3).

Self-reported physical activity measured by the IPAQ showed high activity levels among the patients in this study. During the scoring procedure, 5 participants were excluded from the analysis (due to either missing information regarding duration of physical activity or being an outlier: self-reported total activity >960 min). In total, 8 patients (23%) showed total MET-min values equivalent of low activity, while 77% reported at least moderate activity levels. In terms of exercise intensity, 8 patients reported only walking activity during the last week. The remaining patients performed at least moderate if not vigorous activity. The 12 participants who showed results below the individual age, sex, height and weight-predicted values in the 6MWT also seemed to have impairments in mobility, self-reported physical activity and health-related quality of life. Furthermore, they had higher levels regarding depression and anxiety (Table 4). With respect to the small sample size of *n* = 40, no further statistical evaluation was performed.

**Table 4:** Comparison of the patients below (*n* = 12) the individual age, sex, height and weight-predicted values, with the remaining patients

	6MWT (m)	Age	POMA (points)	TUG (s)	HGST (kg)	HADS (points)	FES (points)	MET-min/week	QOL (points)	Pain (points)
*Patients below their reference values (n* *=12)*										
Mean	419.8	65.8	19.0	12.2	29.8	11.4	25.6	2902.7	57.6	37.5
SD	123.9	12.1	4.8	4.1	10.3	4.9	8.4	2513.1	25.5	29.4
Median	426.0	65.0	19.5	11.5	31	13.0	22.5	1413.0	58.3	33.3
*Remaining patients (n* *=28)*										
Mean	602.8	62.9	25.4	8.3	34.1	10.1	19.3	2791.4	61.0	36.3
SD	83.2	7.3	3.4	1.5	9.7	5.1	3.3	3336.9	20.0	31.4
Median	601.0	62.5	27.0	8.2	34.0	10.0	18.0	1653.0	66.7	33.3

*SD* standard deviation, *m* male, *f* female, *6MWT* 6-min walk test, *POMA* Tinetti performance oriented mobility assessment, *TUG* timed up and go test, *HGST* handgrip strength test of the dominant hand, *HADS* hospital anxiety and depression scale, *FES* falls efficacy scale, *MET* metabolic equivalent of task, *QOL* quality of life

The ORs are presented in Table 5. All ORs showed only very weak evidence of impacting any of the outcomes (OR of 1 contained in the 95% confidence interval). In terms of effect size, an approximation of Cohen’s d was calculated from the OR (Table 6). Among all recorded disease-modifying factors, only stem cell therapy in addition to chemotherapy showed a strong effect on three of the four primary outcome parameters (reducing the odds of a pathological outcome). Oddly, the positive patient characteristics had a positive effect size on not reaching the cut-off in the primary outcomes.

**Table 5:** Odds ratios for pathologic test results

	TUG (s)	POMA (points)	6MWT (m)	HGST (kg)
Rehabilitation	1.07 (0.26; 4.10)	1.48 (0.28; 4.93)	0.73 (0.23; 3.27)	2.26 (0.26; 7.68)
Stem cell therapy	0.10 (0.05; 2.54)	0.17 (0.02; 8.98)	0.11 (0.02; 7.43)	1.19 (0.14; 7.96)
PNP	1.55 (0.30; 4.78)	1.01 (0.24; 4.19)	1.18 (0.24; 3.47)	1.61 (0.23; 6.61)
Smoking	1.57 (0.14; 10.4)	0.43 (0.03; 14.6)	4.37 (0.22; 16.2)	12.4 (0.31; 28.3)

*TUG* timed up and go test, *POMA* performance oriented mobility assessment, *6MWT* 6-min walk test, *HGST* handgrip strength test, *PNP* polyneuropathy

**Table 6:** Effect size on failing an outcome parameter cut-off

	TUG (s)	POMA (points)	6MWT (m)	HGST (kg)
Rehabilitation	−0.02	0.09	−0.07	0.20
Stem cell therapy	−0.57	−0.42	−0.52	0.04
PNP	0.17	0.00	0.04	0.11
Smoking	0.13	−0.20	0.35	0.60

*TUG* Timed up and Go test, *POMA* Performance Oriented Mobility Assessment, *6MWT* 6-minute walk test, *HGST* Handgrip strength test, *PNP* Polyneuropathy

## Discussion

The present study was performed by analyzing members of two self-help groups in Austria. Self-help groups are an essential measure of patient involvement and play an important part in the healthcare system. Members of self-help groups are often well-informed about the management of the disease and highly motivated to participate in regular physical activity. The finding that most patients reported at least moderate to high levels of physical activity supports this observation. Moreover, a substantial amount of study participants reached the performance of the healthy peers in tests of physical performance, such as the 6MWT (*n* = 28, 70%), the TUG test (*n* = 29, 73%), the POMA (*n* = 26, 65%) and handgrip strength test (*n* = 34, 85%). While this performance seems to be underestimated by the patients themselves, they tend to overestimate theispecific risk of falling (FES). This discrepancy is of particular interest as a higher subjective concern of falling can lead in the long run to a vicious circle between impaired physical performance and physical activity. This consequently leads to loss of muscle and bone mass lowering bone density and therefore increasing risk of bone fractures. This is in line with the results as the 12 participants with results in the 6MWT below the individual age, sex, height and weight-predicted values had lower self-reported physical activity and health-related quality of life as well as higher levels of depression and anxiety. Patients with impaired subjective and objective walking performance should therefore be encouraged to at least maintain physical activity levels for as long as possible and perform suitable and supervised individual exercise to improve sensorimotor function, mobility, muscle strength, endurance capacity and independence from others’ help. In comparison, exercise studies with multiple myeloma patients usually exclude patients with pathological fractures or increased risk of fractures [11, 16, 17]. Therefore, a multidisciplinary and interdisciplinary approach as described is necessary to manage the actual condition and to evaluate bone load-bearing capacity of this patient group [11, 18, 19].

Most patients (75%) who did not reach normal values in the 6MWT, TUG, POMA or handgrip strength tests did not reach their goal in at least two of these tests. Data only allow a differentiation between patients who reached the reference values and those who did not. More than half (*n* = 22, 55%) of the patients reported having symptoms of polyneuropathy, which could influence the fear of falling and fall risk in theory; however, the performance in the four objective physical performance tests (6MWT, TUG, POMA, HGST) did not seem to differ between patients with PNP and those without. Overall 20 (50%) patients in the sample of 40 multiple myeloma patients did not reach the reference values in at least 1 of the tests for handgrip strength, exercise capacity (6MWT) and risk of falling (TUG test and POMA), while the other 50% performed above cut-off values in all tests (Table 3). Clinical recommendations for group therapy could safely be given to the latter patient sample, while the former group would be assigned to single therapy sessions. Additionally, a patient’s medical history, clinical examination, laboratory parameters, electrocardiography, echocardiography, exercise testing, spirometry, and radiographic findings are required for planning and prescribing regular physical activity and exercise for patients suffering from multiple myeloma in an interdisciplinary setting [11, 18, 19]. Special clinical features that clinicians have to consider are bone manifestation, hypercalcemia (with risk of cardiac arrhythmia or kidney failure) and monoclonal gammopathy [11, 18, 19]. Additionally, due to the natural history of multiple myeloma with changing clinical status over time, exercise should be performed under supervision of physicians. Besides these objective measures there are also subjective measures, such as pain which was assessed in this study via EORTC QLQ-C30; however, even the patients in the non-fit group (6MWT values below the individual predicted values) reported pain intensity similar to the reference values. Moreover, besides radiographic findings pain is an important factor for evaluation of spinal stability of cancer lesions [33] and pathological fractures [34, 35]. As pain will always be a limiting factor for physical activity and exercise, the symptom pain should be considered when planning exercise groups and treated in a multidisciplinary manner by use of a multimodal approach [19].

None of the recorded patient characteristics (oncological rehabilitation, additional stem cell therapy, polyneuropathy, and smoking) could establish statistical evidence of modifying the outcome of the primary parameters (POMA, TUG, 6MWT, HGST). Stem cell therapy, however, showed a strong effect size on the balance tests and walking distance. A future study on multiple myeloma patients should therefore bolster this finding with greater sample size. Moreover, more confounding variables from the patient’s medical records, such as bone metastases, fractures, precise chemotherapy regimen and a more detailed history of physical therapy should be taken into account.

The present study has some limitations. The majority of the mobility tests were developed for use in geriatric patients in the original context. For the POMA and TUG there are various existing cut-off points, classifications and approaches for the test procedure in the literature but mostly researched in older people [20]. Moreover, due to non-existing reference groups in patients older than 65 years, the oldest participants were classified as if they were 65 years old. Despite the low sample size of *n* = 40 patients, statistical inference was possible to a certain degree; however, a larger sample would have been beneficial and would have allowed a stronger generalizability. When comparing subgroups, however, statistical analysis is less reliable and warrants great caution. Another weakness of the assessment battery was its length, which might have discouraged some participants from participating in this study in the first place. Finally, the recruitment of this study sample was performed via invitations. Therefore, a selection bias regarding motivation to participate is to be expected. Clinical experience shows that self-help group members are especially motivated, educated and active, which is a clear bias towards a generally healthier patient sample. Moreover, assessing fracture risk from the existing patients’ diagnostics, which were incomplete in this respect and not including radiographic assessments during the study protocol made it impossible to categorize the participants into high, moderate, and low fracture risk as a possible basis for more precise recommendations for regular physical activity and exercise in patients with multiple myeloma.

## Conclusion

A substantial number of participants reached the performance of healthy peers in tests of physical performance and reported high levels of physical activity. Nevertheless, they tended to overestimate the specific risk of falling. Patients with notably impaired physical performance might be suitable to perform regular physical activity and exercise in an individual therapy, whereas those with good physical performance are suited for training in exercise groups. To ensure safety and effectiveness of these interventions in patients with multiple myeloma, however, individual contraindications and clinical considerations should be noted in a multiprofessional and interdisciplinary setting.
